# Todani type III: like biliary dilatation with duodenal prolapse—a case report

**DOI:** 10.1186/s12876-022-02278-x

**Published:** 2022-05-04

**Authors:** Yunpeng Meng, Kangli Guo, Yu Jiang, Shaohua Wei

**Affiliations:** 1 The Second Affiliated Hospital of Soochow University, Suzhou, China; 2 Dushu Lake Hospital Affiliated to Soochow University, Suzhou, China

**Keywords:** Biliary dilatation, Duodenal prolapse, Endoscopic ultrasonography

## Abstract

**Background:**

Biliary dilatation is a rare disease involving intrahepatic and extrahepatic biliary tract abnormalities. With the development of imaging technology, an increasing number of special cases have been diagnosed, which poses a challenge to the traditional classification method.

**Case presentation:**

A 50-year-old woman was admitted to the hospital due to right upper quadrant pain for more than 10 days. The patient had previous episodes of similar symptoms, which were relieved after symptomatic treatment at a local community hospital. After the symptoms developed, she underwent a computed tomography scan at the local hospital, which showed biliary dilatation; thus, she was referred to our hospital for further treatment. After admission, her magnetic resonance imaging examination also suggested biliary dilatation, but abnormal signals were found in her duodenum. First, a duodenal diverticulum was considered. Later, endoscopic ultrasonography was conducted, and the results suggested that the dilated biliary tract had herniated into the duodenum. This type of lesion is most closely classified as a Todani type III lesion. The patient finally underwent choledochectomy and Roux‑en‑Y hepaticojejunostomy, and the postoperative pathology was consistent with our preoperative diagnosis. The patient was followed up for approximately 2 years, and no obvious postoperative complications were found.

**Conclusions:**

The manifestations of this case are relatively rare and involve one of the undiscussed categories of the Todani classification system; therefore, this case has certain clinical value. Moreover, there is no report similar to this experience in the previous literature.

## Background

Biliary dilatation is characterized by intrahepatic and extrahepatic biliary tract abnormalities. This disease occurs mainly in Asian populations, with an incidence rate of 1:100, while the incidence rate in Western populations is 1:100,000–1:150,000 [1, 2].Women are more commonly affected than men [3]. The disease was first reported by Vater and Ezler in 1723. Alonso-lej et al. classified the disease into 3 categories for the first time in 1959 and named it choledochal cyst. Todani then classified the disease into 5 types based on the same criteria in 1977, and this standard classification system is currently widely used.

## Case presentation

A 50-year-old female who was otherwise healthy was admitted to the hospital due to a complaint of right upper quadrant abdominal pain for more than 10 days. The patient did not have nausea, vomiting, or fever. The patient’s pain did not improve after rest. Then, she went to the local hospital, where she was initially diagnosed with pancreatitis. She felt slightly better after symptomatic treatment. At the same time, CT revealed choledochal cysts. Therefore, she was admitted to our hospital for further evaluation and treatment. Physical examination showed mild tenderness in the right upper abdomen. There was neither rebounding pain nor jaundice. No hepatosplenomegaly was detected. Her haematology and biochemistry investigations were otherwise unremarkable except for an increased amylase level of 213 U/L. She had no known family history of biliary diseases, and she did not have any chronic diseases.

**Fig. 1:**
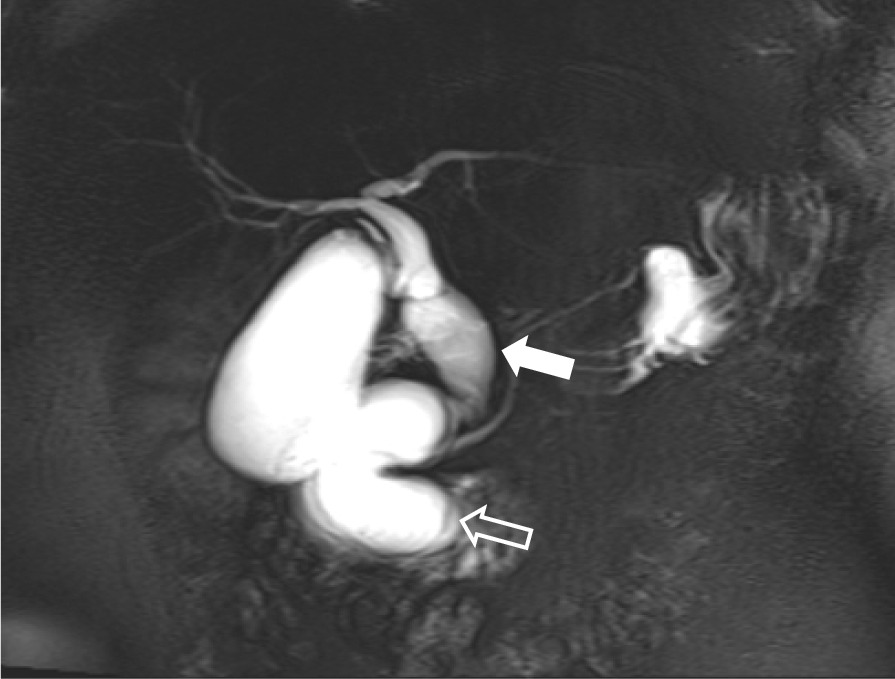
MRCP shows dilation of the common bile duct (arrow), the pancreatic duct orifice is located in the wall of the dilated bile duct, and a filling defect can be seen in the duodenal cavity. At first it was considered a duodenal diverticulum, but later it was confirmed to be a herniated dilated bile duct (open arrow)

**Fig. 2:**
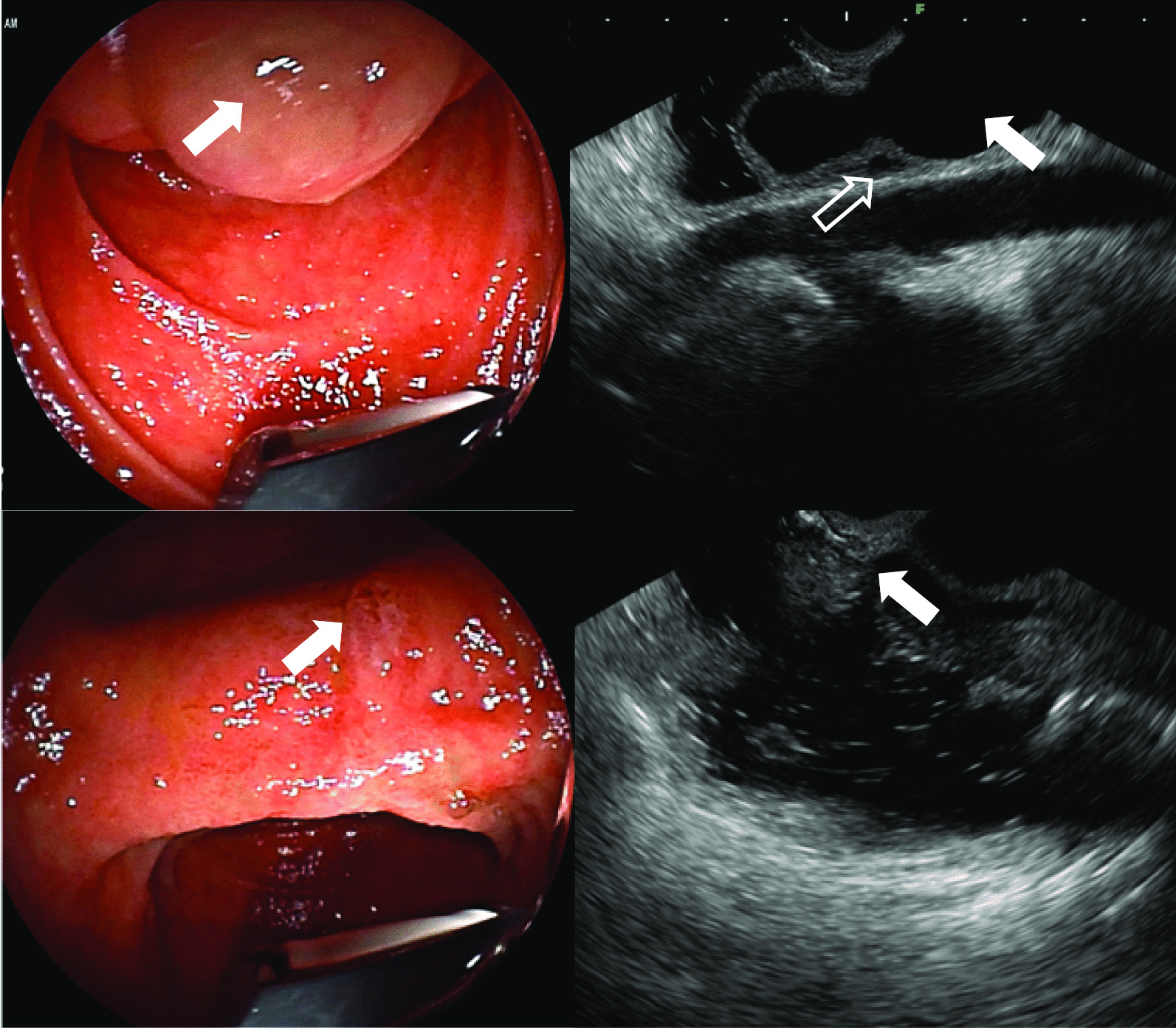
Endoscopic ultrasound shows that the end of the common bile duct expands and herniates into the duodenal cavity (②A arrow). The duodenal papilla located at the wall of the herniated intestinal cavity, which could be returned to its position after inflation (②B arrow). The common bile duct (②C arrow) compresses the pancreatic duct causing its secondary dilation (②C open arrow). No obvious abnormality of duodenal papilla (②D arrow)

Magnetic resonance cholangiopancreatography showed that the common bile duct was thickened and dilated with a diameter of approximately 20 mm. There was no content inside or obvious expansion of the intrahepatic bile duct. The radiologist considered that the descending duodenal diverticulum caused partial obstruction and dilation of the end of the common bile duct (Fig. 1). Therefore, endoscopic ultrasonography (EUS) was conducted, and the findings suggested that the end of the common bile duct (CBD) had expanded and herniated (with a diameter of approximately 17.8 mm) into the duodenum, but the intrahepatic bile duct was not dilated. The duodenal papilla was located on the wall of the herniated intestinal cavity; after inflation, the herniated intestine returned to its normal position (Fig. 2). Therefore, we contacted the radiologist to read the images again and ruled out the possibility of a descending duodenal diverticulum.

**Fig. 3:**
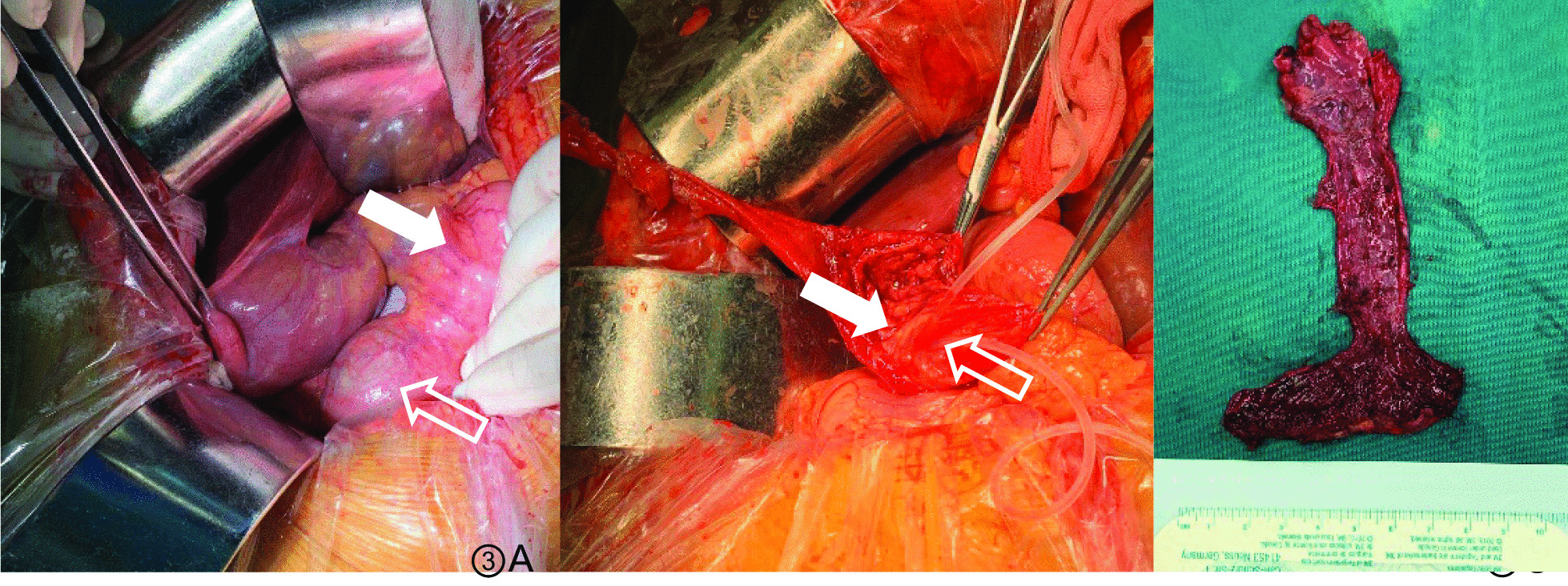
**A** Intraoperative exploration revealed dilation of the common bile duct (arrow), obvious dilation of the terminal common bile duct (open arrow). **B** The dilated bile duct is removed, a supporting drainage tube is placed in the pancreatic duct (arrow) and the duodenal papilla (open arrow), and the remaining cyst wall is sutured with purse-string sutures. **C** The excised specimen

**Fig. 4:**
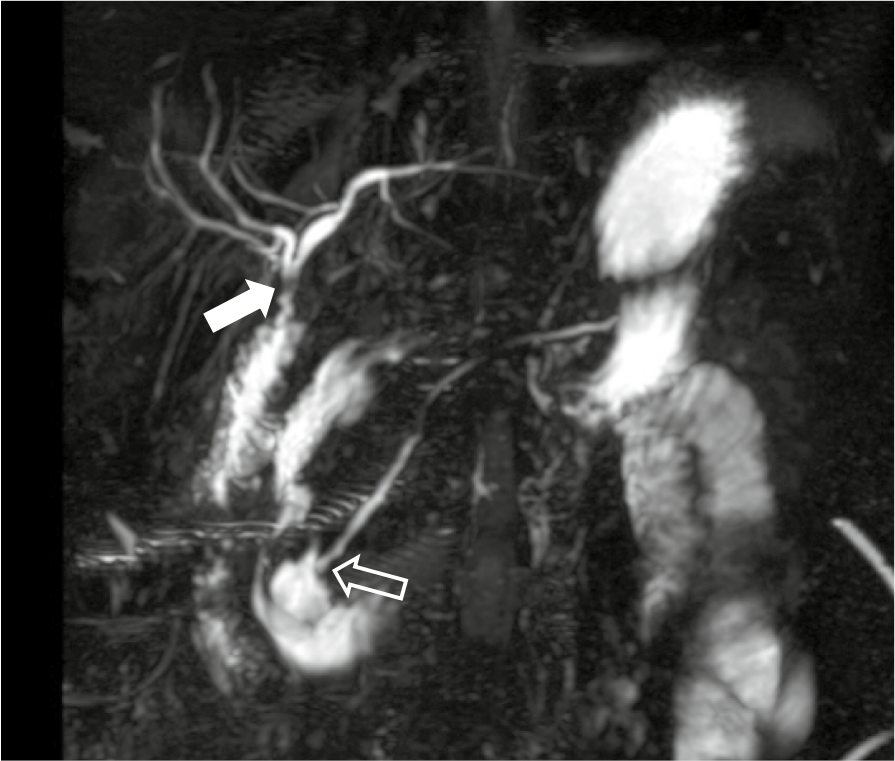
This postoperative MRCP image clearly shows the operation procedure, in which the gallbladder and common bile duct were removed, the common hepatic duct was anastomosed to the jejunum (arrow), and the pancreatic duct was anastomosed to the duodenum (open arrow)

Intraoperative exploration showed obvious biliary dilatation with a maximum diameter of 5 cm, and there were no stones or masses inside the common bile duct (Fig. 3). Therefore, a choledochal cyst resection and Roux-en-Y hepaticojejunostomy were performed (Fig. 4). The postoperative pathological diagnosis revealed chronic cholecystitis with mixed stones and adenomyosis; the common bile duct specimens were consistent with cysts. The patient recovered well after the operation, and was followed up for almost 2 years after discharge, and there were no obvious postoperative complications.

## Discussion

The aetiologies of most of the types of bile duct dilatation are unclear, except for Caroli’s disease, that is, Todani type V, which has been shown to be caused by the mutation of the PKHD1 gene on chromosome 6p12. The most widely accepted aetiological theory is the pancreaticobiliary maljunction (PBM) theory proposed by Babbitt in 1969 [4]. According to this theory, dysplasia of the ventral pancreas (which is formed from the bilobed ventral pancreatic anlagen at 4 weeks of gestation) causes abnormal confluence of the pancreatic and bile ducts at a distance of 1–2 cm from the sphincter of Oddi [5]. Such a common long channel allows for the return of pancreatic juice back into the biliary tract, which leads to increased intrabiliary pressure and possibly to proteolysis of the common bile duct, ultimately leading to bile duct dilatation.

**Fig. 5:**
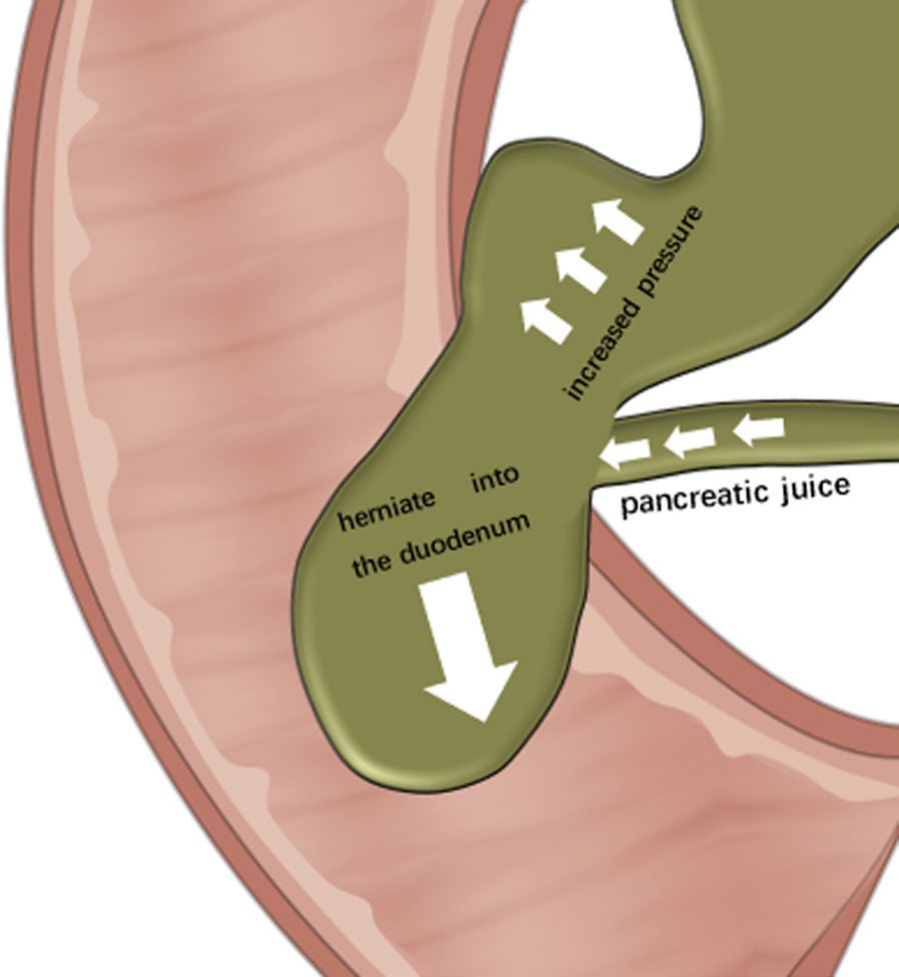
This is a schematic illustration of the possible cause of the patient’s disease. The inflow of pancreatic juice into the bile duct causes the pressure in the dile duct to increase. The long-term sustained high pressure causes the bile duct to herniate into the duodenum through the Oddi sphincter

In this case, the bile and pancreatic ducts were connected in a "P to B" manner. Except for the patient’s elevated amylase, the patient otherwise had no abnormalities in the other examination indicators at the time of admission, and these types of findings are basically consistent with the theory of abnormal biliopancreatic confluence. In addition, the preoperative imaging results and intraoperative findings revealed that the pancreatic duct opened into the dilated bile duct. Therefore, we speculate that the possible cause of the patient's disease was that the pressure of the pancreatic duct was greater than that of the bile duct, so the pancreatic juice that was flowing into the bile duct increased the pressure of the bile duct and caused a bile duct injury, which is the reason for the patient's previous repeated abdominal pain. With the continuous increase in pressure within the bile duct, the bile duct eventually herniated into the duodenum through the Oddi sphincter (Fig. 5). According to the most widely used Todani classification [6] (Table 1), we initially classified this case as type III. However, the dilation of the common bile duct that was observed on ultrasound gastroscopic examination and intraoperative examination did not meet the definition of type III. Todani's definition of type IV bile duct dilatation is rather vague, and the classification does not mention whether the pancreatic segment is included; therefore, it seems inappropriate to define it as type IV. Unfortunately, the patient had no previous imaging examinations when she was symptomatic; therefore, we have no data to compare the current examination results. Nevertheless, we believe that the patient's classification is closer to type III.

**Table 1:** Types of biliary dilatation in the Todani classification

Classification	Anatomic features of the bile duct
Type I	
Type Ia	Cystic dilatation of the common bile duct
Type Ib	Segmental expansion of the common bile duct without an anomalous pancreaticobiliary junction
Type Ic	Diffuse or cylindrical dilatation of the common bile duct with an anomalous pancreaticobiliary junction
Type II	Diverticulum type in the whole extrahepatic duct
Type III	Choledochocele located in the duodenal wall which is frequently associated with an ampullary obstruction
Type IV	
Type IVA	Multiple cysts in both the intrahepatic and extrahepatic bile ducts
Type IVB	Multiple dilatations confined only to the extrahepatic bile duct
Type V	Single or multiple dilatations of the intrahepatic bile duct (Caroli’s disease)

Bile duct dilatation is prone to causing many complications of the pancreas and biliary system, which mainly include neonatal liver fibrosis, adult cholangitis, pancreatitis, and cholecystitis. Long-term pancreatic reflux causes bile duct endothelial metaplasia, which has the potential for malignant transformation. Statistically, 21.6% of patients with biliary dilatation have biliary tract cancer, of which approximately 62.5% of the cases of biliary tract cancer are gallbladder cancer, 4.7% are cases of gallbladder cancer combined with cholangiocarcinoma, and 62.5% are cholangiocarcinoma [5]. Therefore, once biliary dilatation is diagnosed, surgical treatment should be performed as soon as possible, and the dilated bile duct should be completely removed. Approximately 0.7–3% of patients, especially Todani type I and IV patients, develop cholangiocarcinoma after the surgical resection [7]. An incomplete resection of the dilated bile duct and the field effect of the bile duct epithelium can both increase the inherent susceptibility to malignant transformation at the cellular level [8, 9].

Previous studies have shown that Magnetic Resonance Cholangiopancreatography (MRCP) has a sensitivity of up to 96% in the diagnosis of choledochal cysts [10]; however, its reported sensitivity in detecting aberrant pancreaticobiliary junctions has been as low as 53% [11]. In this case, the initial implementation of MRCP suggested that the descending duodenal diverticula caused a partial obstruction and the dilation of the common bile duct. Later, EUS suggested that the biliary dilatation had compressed the pancreatic duct and caused dilation; then, the choledochal had herniated into the duodenum. After discussion with the radiologist, we concluded that the diagnosis obtained by EUS was indeed correct. This shows that when the structure of the biliary disease is confusing, EUS can play a very important role as an auxiliary diagnostic test; because the operator can dynamically and continuously observe the shape of the bile duct and can determine whether the cyst is connected to the CBD during the EUS examination.

Complete surgical excision is the optimal treatment for biliary dilatation. Roux‑en‑Y hepaticojejunostomy and hepatoduodenal anastomosis are the conventional extrahepatic biliary reconstruction methods, among which the most commonly used anastomosis is Roux‑en‑Y hepaticojejunostomy [1, 2, 12]. In this case, because the opening of the pancreatic duct was located in the dilated bile duct, the dilated cyst was not completely removed during the operation, and a purse-string suture was placed on the residual cyst. Therefore, close follow-up after the operation was required to determine if the unresected remnant was cancerous. However, assuming that the bile duct of the pancreatic segment has been completely removed, the bile duct would then have been stripped from the pancreatic parenchyma, and the incidence of postoperative pancreatic leakage would significantly increase. Fortunately, the patient had no postoperative complications during the nearly 2-year follow-up.

In short, for the diagnosis and management of patients with biliary dilatation, a comprehensive and accurate preoperative evaluation should be carried out. According to the patient's condition, a safe and appropriate surgical plan should be selected to avoid complications as much as possible. In addition, patients need to be followed up for a long time.

## Data Availability

All data generated or analysed during this article are included in this published article.

## Abbreviations

BD: Biliary dilatation
CT: Computed tomography
MRI: Magnetic resonance imaging
EUS: Endoscopic ultrasonography
CBD: Common bile duct
PBM: Pancreaticobiliary maljunction
MRCP: Magnetic resonance cholangiopancreatography
